# Investigation of Experimental Factors That Underlie *BRCA1/2* mRNA Isoform Expression Variation: Recommendations for Utilizing Targeted RNA Sequencing to Evaluate Potential Spliceogenic Variants

**DOI:** 10.3389/fonc.2018.00140

**Published:** 2018-05-03

**Authors:** Vanessa L. Lattimore, John F. Pearson, Margaret J. Currie, Amanda B. Spurdle, kConFab Investigators, Bridget A. Robinson, Logan C. Walker

**Affiliations:** ^1^Mackenzie Cancer Research Group, Department of Pathology and Biomedical Science, University of Otago, Christchurch, New Zealand; ^2^Biostatistics and Computational Biology Unit, University of Otago, Christchurch, New Zealand; ^3^Department of Pathology and Biomedical Science, University of Otago, Christchurch, New Zealand; ^4^Genetics and Computational Biology Division, QIMR Berghofer Medical Research Institute, Brisbane, QLD, Australia; ^5^Peter MacCallum Cancer Centre, Melbourne, VIC, Australia; ^6^Canterbury Regional Cancer and Haematology Service, Canterbury District Health Board, Christchurch Hospital, Christchurch, New Zealand

**Keywords:** BRCA1, BRCA2, mRNA, quantitative, splicing, next-generation sequencing, targeted next-generation sequencing, mRNA isoforms

## Abstract

PCR-based RNA splicing assays are commonly used in diagnostic and research settings to assess the potential effects of variants of uncertain clinical significance in *BRCA1* and *BRCA2*. The Evidence-based Network for the Interpretation of Germline Mutant Alleles (ENIGMA) consortium completed a multicentre investigation to evaluate differences in assay design and the integrity of published data, raising a number of methodological questions associated with cell culture conditions and PCR-based protocols. We utilized targeted RNA-seq to re-assess *BRCA1* and *BRCA2* mRNA isoform expression patterns in lymphoblastoid cell lines (LCLs) previously used in the multicentre ENIGMA study. Capture of the targeted cDNA sequences was carried out using 34 *BRCA1* and 28 *BRCA2* oligonucleotides from the Illumina Truseq Targeted RNA Expression platform. Our results show that targeted RNA-seq analysis of LCLs overcomes many of the methodology limitations associated with PCR-based assays leading us to make the following observations and recommendations: (1) technical replicates (*n* > 2) of variant carriers to capture methodology induced variability associated with RNA-seq assays, (2) LCLs can undergo multiple freeze/thaw cycles and can be cultured up to 2 weeks without noticeably influencing isoform expression levels, (3) nonsense-mediated decay inhibitors are essential prior to splicing assays for comprehensive mRNA isoform detection, (4) quantitative assessment of exon:exon junction levels across *BRCA1* and *BRCA2* can help distinguish between normal and aberrant isoform expression patterns. Experimentally derived recommendations from this study will facilitate the application of targeted RNA-seq platforms for the quantitation of *BRCA1* and *BRCA2* mRNA aberrations associated with sequence variants of uncertain clinical significance.

## Introduction

At least 20% of hereditary breast and ovarian cancer cases contain germline pathogenic variants in breast cancer susceptibility genes *BRCA1* (MIM #113705) or *BRCA2* (MIM #600185) (1). Functioning as tumor suppressor genes, *BRCA1* and *BRCA2* repair single and double-stranded breaks in DNA, a process which can be compromised when variants that disrupt pre-mRNA splicing to create aberrant splice isoforms are present (2–4). These variants may directly disrupt splice sites or splicing regulatory regions, such as exonic splicing enhancers or exonic splicing silencers (5). Resulting splicing aberrations, such as major deletion/retention events and frame shifts, can lead to loss of function through the introduction of premature termination codons, leading to non-functional isoforms that are generally destroyed by nonsense-mediated decay (NMD), or *via* the production of truncated proteins (6). In addition, variants located at splicing regulatory regions, such as exonic splicing enhancers, have been shown to significantly alter the abundance of natural *BRCA1/2* isoforms (7, 8). We and others have recently employed next-generation sequencing technologies to explore the expression of mRNA isoforms in *BRCA1* and *BRCA2* variant carriers (9–11). A better understanding of expression level changes that reflect normal variation in *BRCA1/2* splicing patterns between individuals would improve our understanding of isoform regulation for identifying variability that is likely to be of clinical relevance.

In-depth qualitative data published for *BRCA1* and *BRCA2* allows for normally expressed mRNA isoforms to be distinguished more easily from aberrant isoforms (12, 13). The Evidence-based Network for the Interpretation of Germline Mutant Alleles (ENIGMA) consortium developed a 5-tier classification system, which uses mRNA splicing information to help interpret the pathogenicity of possible spliceogenic variants (14). Splicing assays for *BRCA1* and *BRCA2* mRNA isoforms have historically employed a PCR-based qualitative (or semi-quantitative) approach, with only very recent work expanding into a quantitative analysis. To assess key elements for splicing assay design and the integrity of published splicing data, a multicentre quality control investigation was conducted by the ENIGMA Splicing Working Group (15). This study highlighted the need to standardize splicing protocols between laboratories after raising a number of methodological issues associated with current PCR-based protocols, including (1) primer design that encompasses only a subset of the *BRCA1* and *BRCA2* exons, (2) non-standardized use of NMD inhibitors, (3) isoforms infrequently confirmed by sequencing, and (4) a qualitative or semi-quantitative approach to assess mRNA expression patterns, as opposed to quantitative assessment (14, 15). Although this study demonstrated variation in analytical sensitivity between samples, and the same sample between participating laboratories, the impact of underlying experimental factors remained unclear.

Targeted RNA-seq technologies potentially address many of the difficulties currently associated with PCR-based assays. For example, RNA-seq platforms enable detection of mRNA isoforms both qualitatively and quantitatively (16, 17), thus producing comprehensive transcript profiles across the entire gene(s). Assessment of the analytical sensitivity of targeted RNA-seq to both qualitatively and quantitatively measure *BRCA1/2* isoform expression in relation to experimental factors would provide a deeper understanding of the sources of mRNA isoform variation in these genes, but has yet to be evaluated.

In this study, we carried out targeted RNA-seq to assess *BRCA1/2* mRNA isoform expression patterns in lymphoblastoid cell lines (LCLs) previously utilized by the ENIGMA-led multicentre study (15). We describe a comprehensive assessment of naturally and aberrantly occurring *BRCA1* and *BRCA2* mRNA isoforms in relation to experimental factors. Our results show that quantitation of relative levels of naturally occurring transcripts is not significantly impacted by key elements of cell-storage and culture protocols, with the possible exception of NMD-inhibition. We provide recommendations for future use of targeted RNA-seq for the analysis of variants that may disrupt RNA splicing.

## Materials and Methods

This study was approved by the Southern Health and Disability Ethics Committee (12/STH/44).

### Samples

27 LCLs derived from 17 *BRCA1* or *BRCA2* rare variant carriers, and 10 healthy controls (Figure S1 and Table S1 in Supplementary Material) were obtained from Kathleen Cuningham Consortium for Research into Familial Breast Cancer. Eighteen of the cell lines (sample IDs LCL1–8 and LCL18–27) were previously used in a multi-center methods splicing analysis study coordinated through the ENIGMA consortium (15).

Variants included in this study are referred to by the recommended Human Genome Variation Society^1^ nomenclature, including use of A in the ATG translation initiation codon to start the nucleotide numbering for *BRCA*1 (GenBank accession—NM_007294.3) and *BRCA2* (GenBank accession—NM_000059.3) (18).

Cell lines were cultured in RPMI 1640 media, with fetal calf serum (10%) and penicillin–streptomycin (1%), while incubated at 37°C in a 5% CO_2_ atmosphere. RNA was isolated from cycloheximide treated (4 h 100 µg/mL) and untreated cells using the Qiagen RNeasy Mini Kit. RNA was reverse transcribed into cDNA using Superscript III First Strand Synthesis System (Invitrogen), according to the manufacturer’s instructions.

### RNA-seq Analysis

Targeted RNA-seq was undertaken on all cycloheximide treated and untreated LCLs using the Illumina Truseq Targeted RNA Expression kit. The targeted sequencing assay was custom designed in Illumina’s design studio using 34 *BRCA1* and 28 *BRCA2* oligonucleotides chosen from a database of validated predesigned probes (Tables S2 and S3 and Figures S2 and S3 in Supplementary Material). Capture of the targeted cDNA sequences was performed according to the manufacturer’s specifications (Figure S4 in Supplementary Material). Sequence analysis was carried out using the Illumina MiSeq platform.

Whole transcriptome sequencing analysis was also carried out for one control (LCL24). Using these data, *BRCA1* and *BRCA2* mRNA isoform splicing expression patterns were also obtained. RNA from LCL24 was sequenced with and without cycloheximide treatment on a HiSeq2000 using the Truseq^®^ Stranded mRNA kit (Illumina).

#### Read Mapping and Processing

Targeted RNA-seq and whole transcriptome read mapping was undertaken as previously described (19). Briefly, the Homo_sapiens.GRCh37.72 reference genome was downloaded from Ensembl^2^ and the chromosomes arranged into lexicographic order prior to mapping. Sequence reads were mapped using the two pass approach of the Spliced Transcripts Alignment to a Reference (STAR) aligner using the default settings unless specified otherwise (20). Maximum intron length was set to 100,000 nucleotides to accommodate for splice junctions that span the length of each gene. Detected splice junctions for each sample were extracted from STAR’s SJ.out file for further analysis. Sample-specific read counts are listed in Table S4 in Supplementary Material.

#### Targeted RNA-seq Data Analysis: Normalization

**Figure 1:**
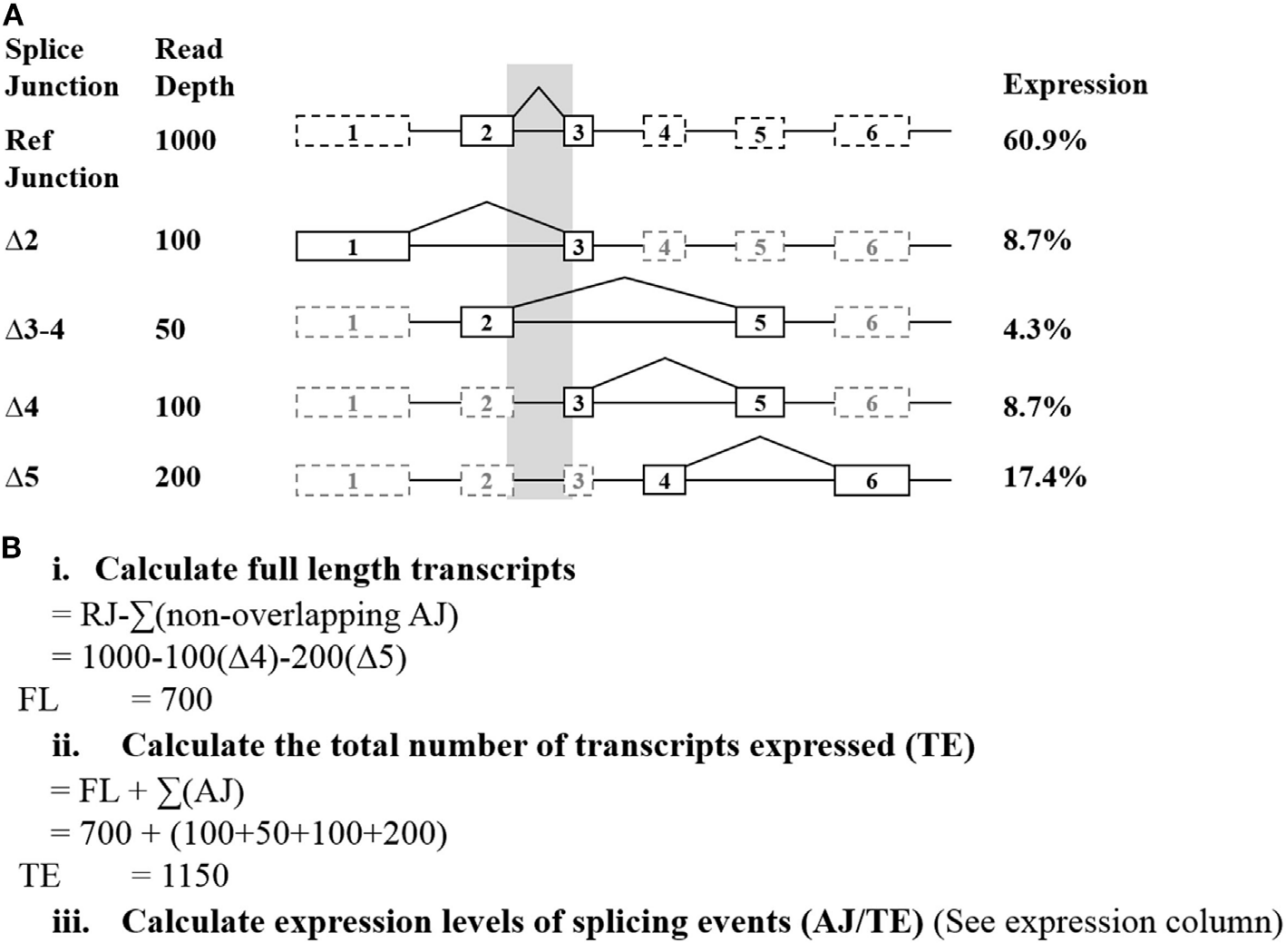
Exemplar of how the relative expression of each splice junction was calculated from targeted RNA-seq data. **(A)** The shaded region indicates the alternative splicing events excluded from the full-length calculations. Solid lines indicate the exons directly involved with an exon skipping event. **(B)** Calculations used to determine the relative expression of each detected junction. Abbreviations: AJ, alternate junction; RJ, reference junction.

Raw read counts of all alternative splicing events were normalized to measure the relative number of individual spliced exon/exon junctions in each sample. This was achieved by calculating the read depth of the full-length transcript (Figure 1). To normalize read depth, the total read count between two adjacent exons (exons 2 and 3 in *BRCA1* and *BRCA2*, respectively) was used as the reference junction (RJ) (hereon referred to as “reference junction”) to calculate the relative expression of the full-length and alternative mRNA transcripts for both *BRCA1* and *BRCA2*, respectively. To achieve this, the sum of all non-overlapping exon 2–3 alternative splicing events was deducted from the RJ for each gene independently. This leaves a proportion of reads that solely represent the full-length transcript (Figure 1Bi). To determine the relative proportion of each splice junction, the total number of reads for each sample was first calculated. This consisted of the sum of the reads encompassing the alternative splicing events together and the previously calculated full-length transcript (using RJ exons 2–3, see above) (Figure 1Bii). The relative proportion of the individual isoforms in each sample is determined by dividing its respective read count by the total number of reads for that sample (Figure 1Biii). These expression values were incorporated into a comparative expression analysis, using the mean and SE (95%) of each isoform across the controls. This approach does not account for the possibility that some of the detected alternative splicing events may occur concurrently. Relative expression ranges were calculated based on the criteria that at least two control samples expressed the transcript with more than 10 reads, each sample was represented by more than 10,000 reads per gene, and each sample expressed at least two minor transcripts for the studied gene.

Alternative events were excluded from these calculations if they had questionable probe efficiency (such as was observed for *BRCA1* Δ9–10) or were common NAG events (21). These are shown to commonly co-occur with the other events detected, and so are not deducted as separate alternative splicing events when calculating the full-length transcript. The resulting proportions were compared using complementary log–log confidence intervals (22).

### Quantitative PCR (qPCR) Validation of Splicing Events

The expression levels observed for the *BRCA1* exon 10–11, Δ10, and Δ9–10 junctions were assessed using Taqman qPCR assays and Roche LightCycler^®^ 480 platform. Primers were designed to encompass each targeted junction type, with the corresponding probe spanning the junction (Table S5 in Supplementary Material). All qPCR assays were carried out in triplicate.

## Results

### Identification of BRCA1 and BRCA2 Isoforms From Targeted RNA-seq Data

Using targeted RNA-seq, we detected 40 *BRCA1* and 17 *BRCA2* alternate isoforms in LCLs from non-variant carrier controls cultured with or without a NMD inhibitor (Tables S6–S7 in Supplementary Material). These include 25/63 of the *BRCA1* isoforms identified by Colombo et al. (12) and/or Davy et al. (10) (Table S6 in Supplementary Material), in addition to 5/22 *BRCA2* isoforms identified by Fackenthal et al. (13) (Table S7 in Supplementary Material). In addition to these, six naturally occurring *BRCA1* isoforms previously reported by Colombo et al. (12) were detected in variant carriers. Targeted RNA-seq also detected 13 *BRCA1* and 11 *BRCA2* isoforms that have not been reported previously in healthy controls. The novel transcripts identified here increases the total number of *BRCA1* and *BRCA2* splicing events observed in control samples to 70 and 34, respectively.

Of the previously reported naturally occurring isoforms not detected in *BRCA1* and *BRCA2* using targeted RNA-seq (12, 13), the majority (28/32 in *BRCA1* and 13/17 in *BRCA2*) were due to target probe placement (restricted to the options predesigned by Illumina), while the remainder (11/32 *BRCA1* and 4/17 *BRCA2*) were presumably not expressed at levels that were detectable in our cell lines (Tables S6 and S7 in Supplementary Material). From our observations, and those reported in previous publications (12), all *BRCA1* mRNA exons have been shown to be spliced out in at least one naturally occurring alternative mRNA transcript. By contrast, six *BRCA2* exons (8, 14, 21, and 24–27) were not found to be involved in an exon skipping event in this study or by Fackenthal et al. (13). Nineteen alternative transcripts (11 *BRCA1* and eight *BRCA2)* were detected solely in the samples treated with a NMD inhibitor (Tables S6–S9 in Supplementary Material). Of the 19, 14 are out of frame.

To assess the targeted RNA-seq method for evaluating transcript profiles in rare variant carriers (LCL1–8), we compared our data with those previously reported from the PCR-based ENIGMA multicentre study (15) (Table S10 in Supplementary Material). In this study, a total of 37 *BRCA1* and 11 *BRCA2* alternative splicing events were identified by targeted RNA-seq in addition to those detected by reverse transcriptase-PCR (RT-PCR) from the multicentre study (Table S10 in Supplementary Material). We found that 33/37 *BRCA1* and all 11 *BRCA2* of these events fell outside the region targeted by the RT-PCR assays. By comparison, 26 *BRCA1* and 12 *BRCA2* splicing events were exclusively detected in these samples in the multicentre study. However, 21 of these events were not detected by the Truseq Targeted RNA Expression platform due to the absence of probes targeting those regions, while 11 were due to the events involving multiple separate regions, which are not detectable together using this platform. Of the remaining six events not detected by Targeted RNA-seq (*BRCA1*, Δ5–6, Δ9, Δ9–11, Δ9–12, Δ11–12, Δ22), three (Δ9, Δ22, and Δ9–11) were respectively identified by three laboratories, which always included the two laboratories utilizing capillary electrophoresis for detection. Further to this, *BRCA1* Δ5–6 was identified solely by laboratories utilizing capillary electrophoresis, which was the most sensitive detection method used in the multi-center study (15).

Targeted RNA-seq identified another five splicing events described by Whiley et al. (15) that were solely present in four out of the eight LCLs each carrying a known spliceogenic rare variant (Table S10 in Supplementary Material, *BRCA1* c.671−2A>G − Δ9–11 and Δ10–11; *BRCA1* c.5467+5G>C − Δ23; *BRCA2* c.8632+1G>A − Δ19–20; *BRCA2* c.9501+3A>T − Δ25). In contrast to the multicentre study, targeted RNA-seq was unable to detect the Δ5 event associated with the pathogenic variant *BRCA1* c.135−1G>T, likely as a result of a low read count (Table S10 in Supplementary Material). These data further highlight the complexity associated with detection and interpretation of *BRCA1* and *BRCA2* splicing patterns when assays are designed across the whole transcript.

### Quantitative Assessment of BRCA1 and BRCA2 Transcripts

**Figure 2:**
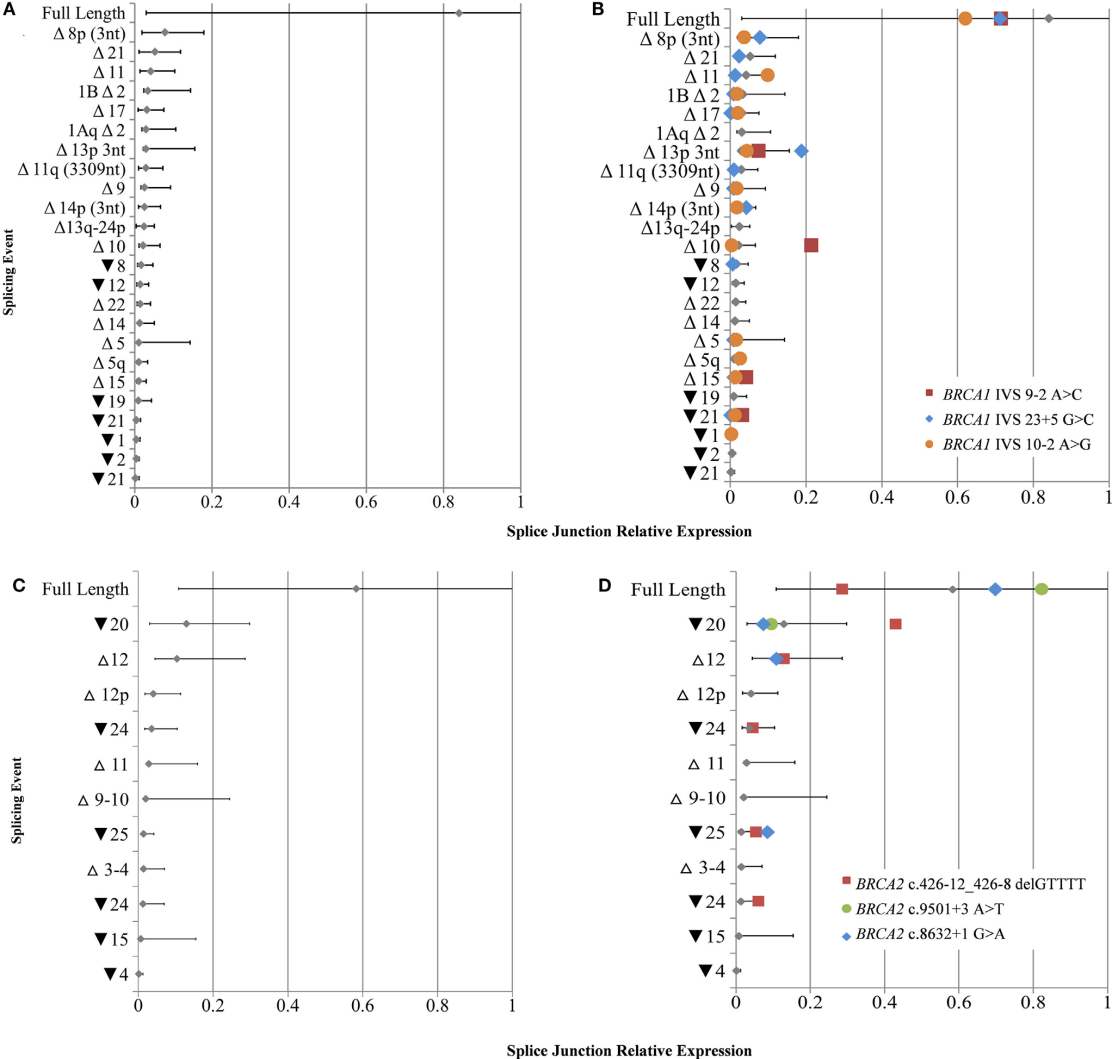
Relative expression of *BRCA1* and *BRCA2* mRNA isoforms in rare variant samples compared to controls. **(A)** Natural expression ranges of mRNA splice isoforms calculated from lymphoblastoid cell lines (LCLs) not containing any known spliceogenic variants in *BRCA1* **(A)** and *BRCA2* **(C)**. Colored symbols overlaid indicate the relative mRNA isoform expression in LCLs containing known *BRCA1* **(B)** or *BRCA2* **(D)** splice disrupting variants. Only mRNA splice isoforms that were detected by more than 10 reads in at least two controls were included. Mean and upper and lower limits shown for each isoform [SE (95%)].

To derive quantitative information from the targeted RNA-seq data, we separately calculated the relative expression range for *BRCA1* (*n* = 25) and *BRCA2* (*n* = 14) transcripts from the study LCLs that did not contain known splice disrupting variants in the respective gene assayed. The number of alternative splicing events detectable across multiple LCLs was double in cycloheximide treated cell lines compared to that found in non-treated cells (Figures 2A,B), thus the following quantitative data corresponds to treated cells only. A correlation was observed between the number of detected alternative events and the total read count per sample for *BRCA1* (*R*^2^ = 0.68) and *BRCA2* (*R*^2^ = 0.69) (Figure S5 in Supplementary Material). The questionable probe efficiency observed for alternative event *BRCA1* Δ9–10 using targeted RNA-seq was confirmed with qPCR to be overinflated (Table S11 in Supplementary Material) and so was excluded from the normalization calculations.

The full-length transcripts were found to be the most highly expressed mRNA isoforms for both *BRCA1* and *BRCA2* when comparing the relative levels of all mRNA isoforms expressed for each gene, while they also had the greatest expression variability (Figure 2). No *BRCA1* splicing events were expressed above 20% of the total number of detected transcripts, whereas the expression ranges of *BRCA2* Δ9–10, Δ12, and ▾20 exceeded this level.

Despite a high mRNA expression variability detected for natural isoforms, results still highlighted notable isoform expression differences between variant carrier and control LCLs not measured by PCR-based methods in the ENIGMA multi-center study (15). The most significant difference was for Δ10 in LCL5 (*BRCA1* c.[594−2A>C; 641A>G]), which expressed an 8.8-fold increase compared to controls. The isoforms Δ15 and ▾21 were also upregulated in this sample (2.8- and 5.0-fold increase, respectively). Expression differences were also found for ▾25 (4.5-fold increase) for LCL8 (*BRCA2* c.8632+1G>A); ▾20 (2.3-fold increase) and ▾25 (2.4-fold increase) for LCL1 (*BRCA2* c.426-12_426-_8delGTTTT); and ▾21 and Δ11 (1.3-fold increase) for LCL4 (*BRCA1* c.671−2A>G) (Figures 2B,D).

To compare targeted RNA-seq and qPCR-derived expression levels, the relative expression of *BRCA1* Δ9–10, Δ10, and the exon 10–11 junction was calculated using a RJ (*BRCA1* exons 2–3) in LCL5 (*BRCA1* c.594−2A>C) and compared to controls. Consistent with recent findings (9), both targeted RNA-seq and qPCR assays measured a significant increase in expression levels of Δ10 (28.688-fold_RNA-seq_ – 16.262-fold_qPCR_), similar levels of Δ9–10 (0.869-fold_RNA-seq_ – 1.150-fold_qPCR_), and reduced 10–11 junction levels (0.718-fold_RNA-seq_ – 0.427-fold_qPCR_) in LCL5 compared to the controls (Table S11 in Supplementary Material).

### The Effect of LCL Storage and Culture Conditions on BRCA1 and BRCA2 mRNA Isoform Expression

**Figure 3:**
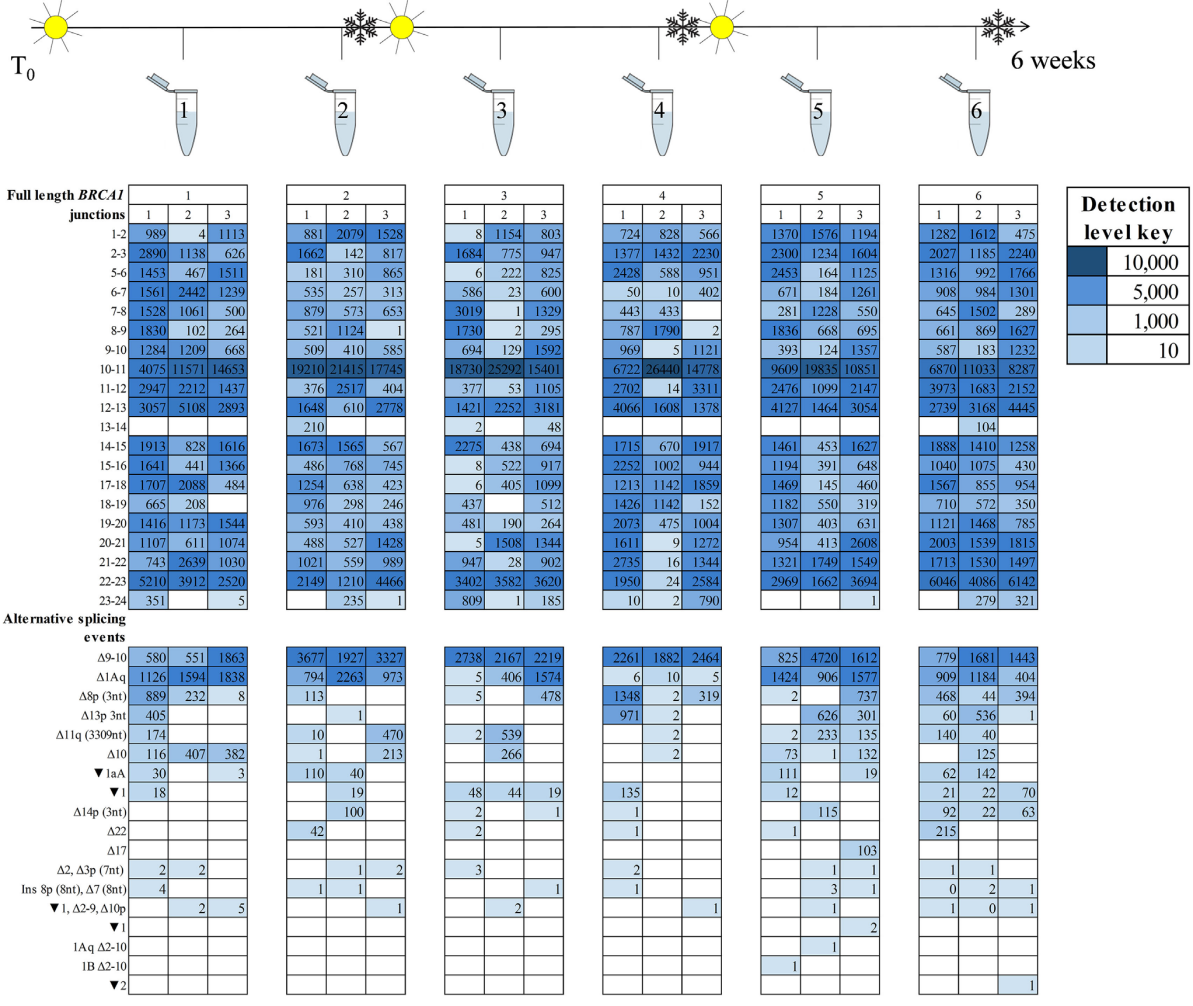
*BRCA1* mRNA isoforms detected at six time points in an lymphoblastoid cell line (sample #7, Table S1 in Supplementary Material) treated with an nonsense-mediated decay inhibitor. A freeze–thaw process was undertaken after time points two and four. Three technical replicates are listed under each time point.

A key observation from the ENIGMA multicentre study was the variability in *BRCA1* and *BRCA2* isoform detection between laboratories using different cell processing and assay protocols (15). However, it was unclear whether these inter-laboratory differences are due to untested aspects of the laboratory protocol. To explore this possibility, we assessed the effect of cell culture and storage conditions on *BRCA1* and *BRCA2* isoform expression in RNA extracted at six time points from LCL7 with fortnightly freeze/thaw cycles. RNA was sequenced using targeted RNA-seq with technical triplicates (Figure 3).

**Figure 4:**
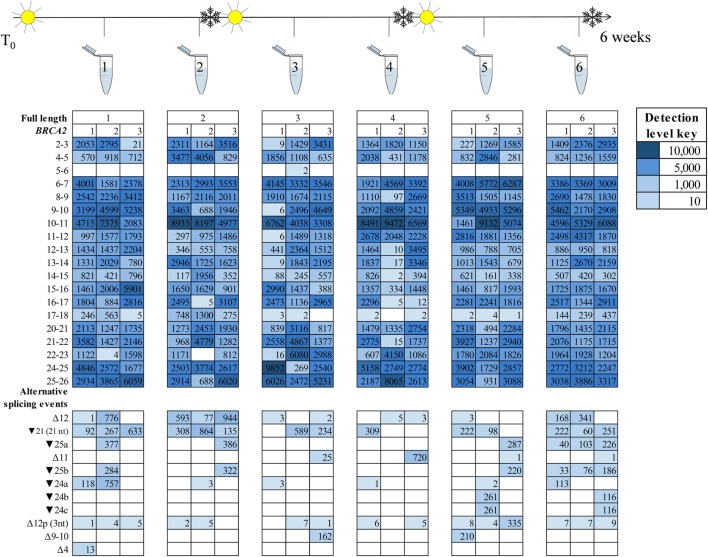
*BRCA2* mRNA isoforms detected at six time points in an lymphoblastoid cell line (sample #7, Table S1 in Supplementary Material) treated with an nonsense-mediated decay inhibitor. A freeze–thaw process was undertaken after time points two and four. Three technical replicates are listed under each time point.

Detection of the more prominently expressed alternative splicing events (for example, *BRCA1* Δ9–10 and Δ1Aq) was more consistent across time points and between technical replicates than it was for the minor events (Figures 3 and 4). Testing for significant isoform expression differences by time point using the linear model found no consistent effect for *BRCA1* and *BRCA2* in either NMD inhibitor treated or untreated samples (Figures S6–S9 in Supplementary Material). Together these results suggest that expression variability does exist at the intra-laboratory level and this variability is greatest for mRNA isoforms detected at low levels, such as the samples not treated with NMD inhibitors (Figures 3 and 4; Figures S10 and S11 in Supplementary Material). However, there was no evidence for systematic effects relating to the number of freeze/thaw storage cycles or whether the cells have been analyzed after 1 or 2 weeks growth.

## Discussion

*BRCA1* and *BRCA2* mRNA splicing assays are often carried out in a diagnostic and research setting to assess the effects of variants of uncertain clinical significance. To date, PCR-based mRNA splicing assays have been the method of choice to assess mRNA transcripts qualitatively. While genetic variation has been suggested to induce abnormal isoform expression changes (23), such aberrations are not easy to detect using non-quantitative or semi-quantitative techniques. Here, we have utilized a targeted RNA-seq approach to qualitatively and quantitatively assess the expression profile of *BRCA1* and *BRCA2* mRNA isoforms in LCLs previously assayed in a multicentre study (15). This whole-gene approach is much more comprehensive than traditional PCR-based splicing assays, as evidenced by the detection of 55 mRNA isoforms (30 known and 25 novel) using only a small fraction of the samples used for the reported catalog of 80 *BRCA1*/*2* isoforms (12, 13). Several transcripts were not detectible due to limitations with the capture design and/or reduced sensitivity, however, lowly expressed *BRCA1* and *BRCA2* isoforms that have not been identified by previous studies were able to be detected using this platform (12, 13). Additional splicing events may be present, but at very low levels, requiring a much higher sequencing depth for detection. Further work is required to determine if such transcripts are clinically important, and so establish whether their detection is required for an understanding of breast cancer risk.

The targeted RNA-seq platform utilized in this work was able to overcome previously reported limitations of PCR-based assays (14, 15) by providing multiple exon coverage across *BRCA1* or *BRCA2* for each assay, sequence confirmation of splicing event, and quantitative assessment of isoform expression patterns. Moreover, our study demonstrated that the use of NMD inhibitors is important for obtaining detectable levels of full length and alternative splicing events using the Illumina Truseq Targeted RNA Expression platform.

We show the utility of targeted RNA-seq to quantitatively identify previously reported upregulated splicing events, such as Δ10 (*BRCA1* c.[594−2A>C; 641A>G]) and Δ11 (*BRCA1* c.671−2A>G) (Figure 2), while the expression levels of many of the other isoforms in the variant carriers were within the range seen in controls. Our study also identified higher levels of Δ15 and ▾21 for LCL4 (*BRCA1*c.[594−2A>C; 641A>G]) than expected in controls, which have not previously been reported, likely because these small differences are not easily observable with semi-quantitative technologies. However, these changes are not associated with pathogenicity in *BRCA1* c.[594−2A>C; 641A>G] carriers (9), and any association between the variant and each splicing event remains unclear. It is possible that future research with additional control samples may show that these small expression changes are within the natural expression range. Interestingly, the relative expression ranges observed for all alternative events appeared to be more tightly regulated in *BRCA1* than *BRCA2*. While they do not appear to overlap any important domains, three *BRCA2* mRNA isoforms were expressed at greater levels than those associated with *BRCA1* (Figure 2). These differences suggest that greater variability in expression for some *BRCA2* isoforms is tolerated in LCLs, however, further research is required to established *BRCA1* and *BRCA2* isoform expression patterns in cancer specific tissue, such as normal breast and ovarian epithelia.

Splicing data from this study, in addition to those from recent reports (10–13), show that every exon in *BRCA1* and 20/27 exons for *BRCA2* are skipped in at least one natural isoform. This highlights how quantitative assessment of aberrant splicing would be very beneficial in these highly variable genes as it would provide a more comprehensive detection method of the splicing changes present. It also suggests that seven *BRCA2* exons are likely to be highly conserved, so any changes involving these exons are likely to be detrimental in the cell.

To identify technical factors that also contribute *BRCA1* and *BRCA2* mRNA expression differences between samples, we assessed the effect of cell culture and storage conditions on *BRCA1* and *BRCA2* isoform expression across the six experimental time points. Our results showed variability in the isoforms expressed at any given time, irrespective of the number of storage events and culture time. Moreover, variability is greater when RNA-seq assays generate relatively low number of sequence reads (Figures 3 and 4; Table S12 in Supplementary Material).

Targeted RNA-seq platform utilize short fragmented library reads to detect mRNA splicing events. Such platforms are, therefore, limited in their ability to determine whether multiple events occur on the same transcript. Recently, we carried out a study using the MinION™ (Oxford Nanopore Technologies, Oxford, UK) long read sequencer and obtained whole transcript information for *BRCA1* in a normal sample, which showed evidence of co-occurring splice events in *BRCA1* (24). Further work involving long read sequencing of *BRCA1* and *BRCA2* would help to further distinguish transcript exon structure regarding all deletion and retention events for variant carriers. Results from such research will also accurately define out-of-frame transcripts which are prone to NMD.

Here, we utilize targeted RNA-seq technology to provide a comprehensive review and quantitative assessment of naturally occurring mRNA isoforms in *BRCA1* and *BRCA2*. While qualitative analysis alone has been assumed to be sufficient for identifying aberrant events, the more quantitative RNA-seq offers improvements to the accuracy and capabilities of PCR-based assays, overcoming many of the detection limitations presented by the earlier technologies. These results lead us to make the following recommendations: (1) technical replicates (*n* > 2) of the variant carrier are necessary to capture methodology induced variability associated with RNA-seq assays, (2) LCLs can undergo multiple freeze/thaw cycles and can be cultured up to 2 weeks without noticeably influencing isoform expression levels, (3) NMD inhibitors are essential prior to splicing assays for comprehensive mRNA isoform detection, (4) quantitative assessment of exon:exon junction levels across *BRCA1* and *BRCA2* can help distinguish between normal and aberrant isoform expression patterns. While advances in probe design, and possibly the uptake of long read sequencers, are essential to allow detection of all expressed mRNA isoforms using this platform, the decreasing costs of RNA-seq technology, alongside an increasing understanding of bioinformatics capabilities, will likely increase the progression away from PCR-based assessment of gene expression, as evidenced by recent work by Davy et al. (10). In addition, the advanced capabilities of RNA-seq promise to aid in evaluating the clinical significance of variants in *BRCA1* and *BRCA2*, but further exploration is required to determine whether these variants are influencing expression. Quantitative assessment of *BRCA1/2* isoforms in a greater number of control samples using other RNA-seq platforms will further improve our understanding of “normal” expression and provide an invaluable reference for establishing the occurrence of aberrant splicing when assessing genetic variants. Furthermore, careful assay design will be crucial for obtaining data across the gene, thus enabling an interpretation of splicing changes for the entire transcript as opposed to selected regions.

## Author Contributions

Conception or design of the work—VL, LW, JP, BR, MC, and AS. Resources—kI and LW. Data collection and drafting the article—VL and LW. Data analysis and interpretation—VL, JP, and LW. Critical revision of the article and final approval of the version to be published—all authors.

## Conflict of Interest Statement

The authors declare that the research was conducted in the absence of any commercial or financial relationships that could be construed as a potential conflict of interest. The handling Editor declared past co-authorship with the authors AS and LW.

## Footnotes

^1^http://www.hgvs.org (Accessed: January 15, 2016).

^2^http://ftp.ensembl.org/pub/release-72/fasta/homo_sapiens/dna/Homo_sapiens.GRCh37.72.dna.primary_assembly.fa.gz (Accessed: July 1, 2014).
