# Supplementary figures and images for: Investigation of Experimental Factors That Underlie BRCA1/2 mRNA Isoform Expression Variation: Recommendations for Utilizing Targeted RNA Sequencing to Evaluate Potential Spliceogenic Variants
Source: Front Oncol. 2018 May 3;8:140. doi: 10.3389/fonc.2018.00140 (PMC5943536; doi:10.3389/fonc.2018.00140)

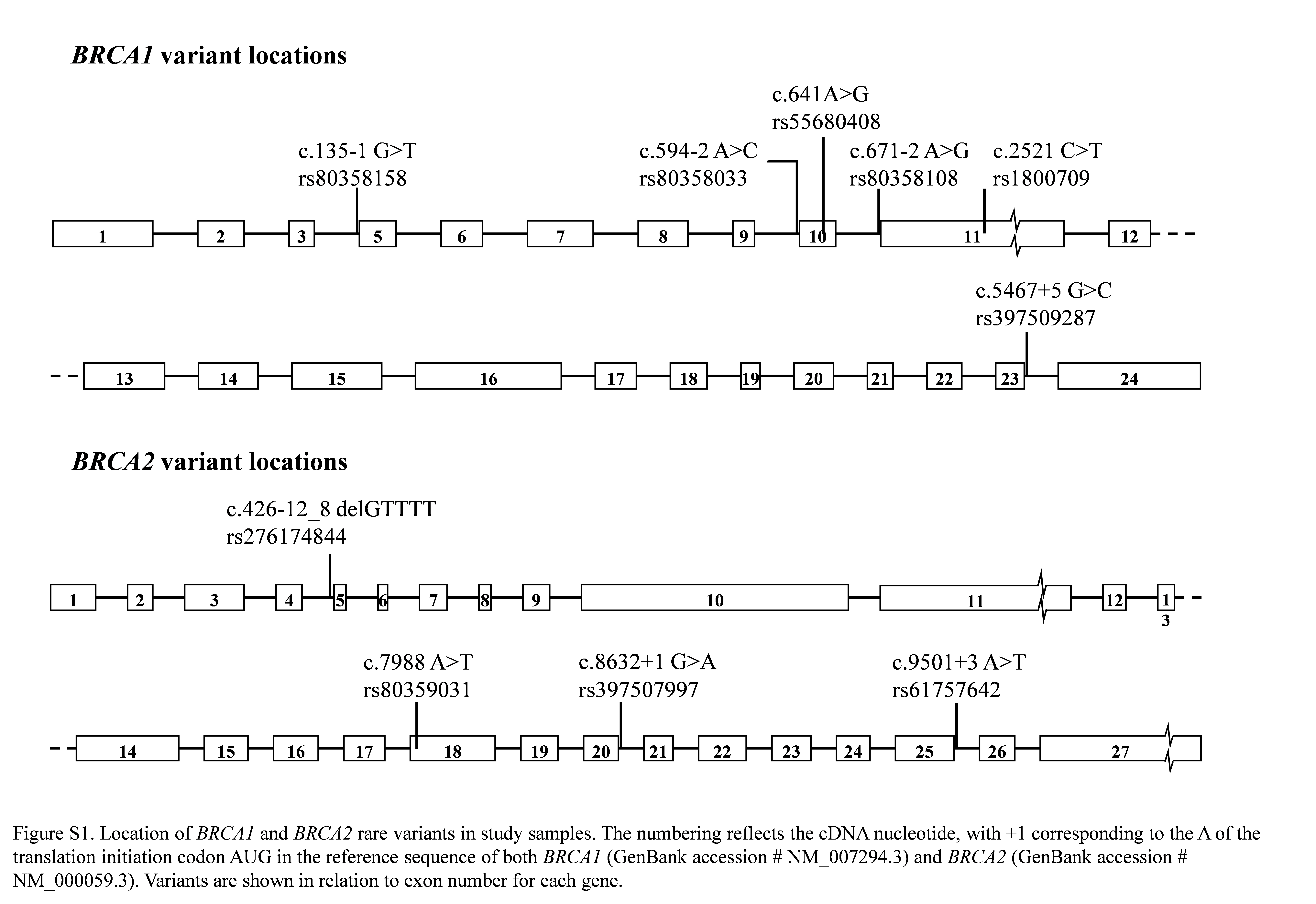

Supplement: Supplementary file 1 [file image_1.tif]

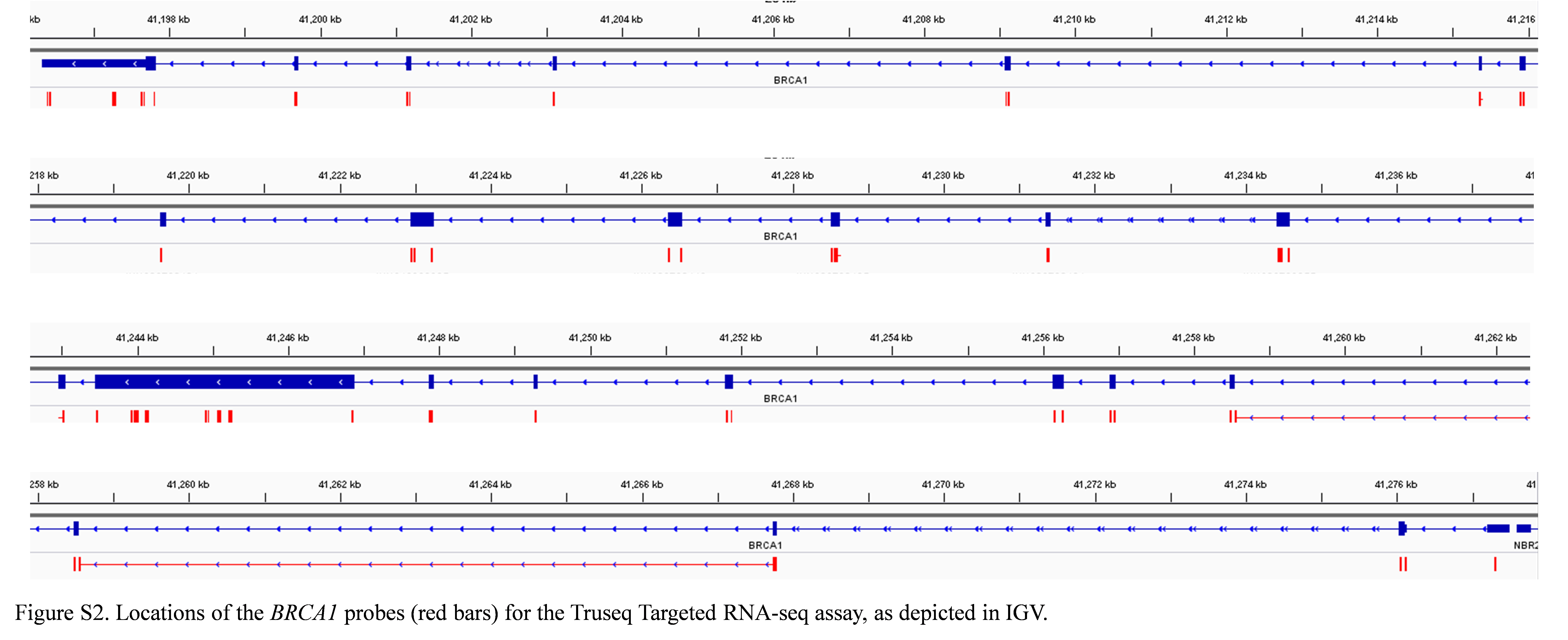

Supplement: Supplementary file 2 [file image_2.tif]

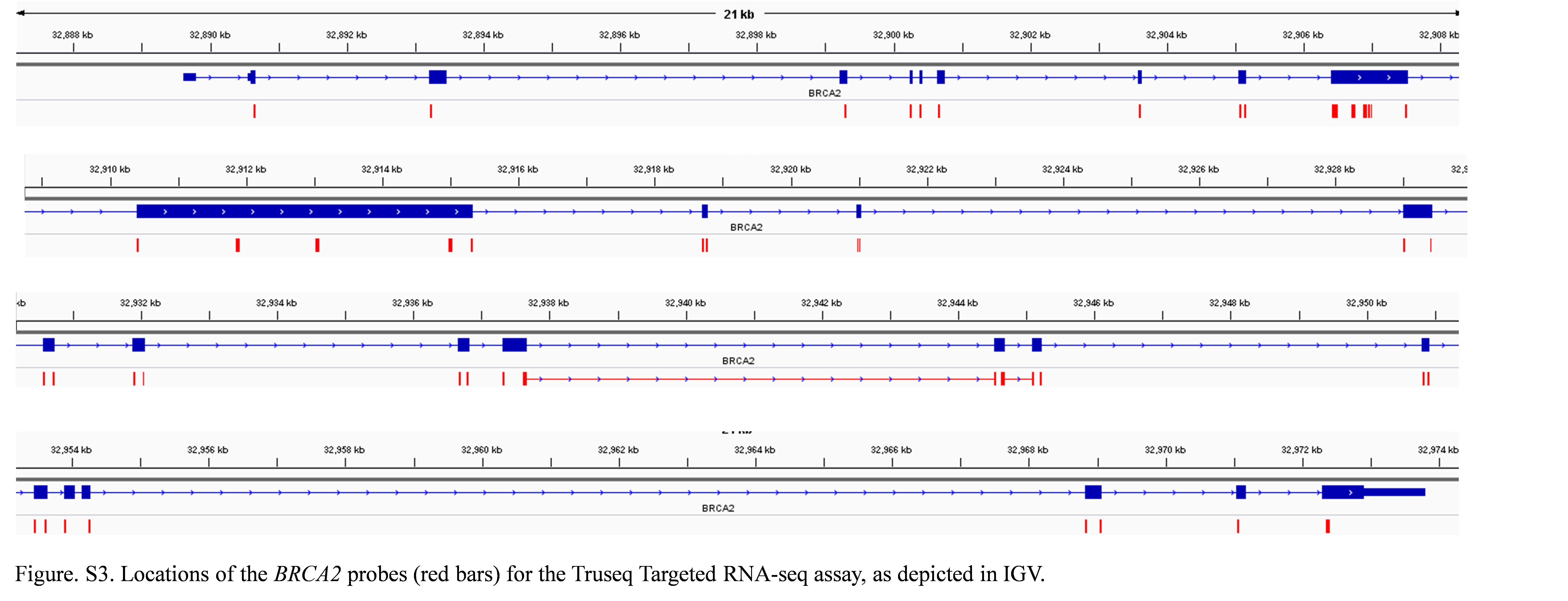

Supplement: Supplementary file 3 [file image_3.tif]

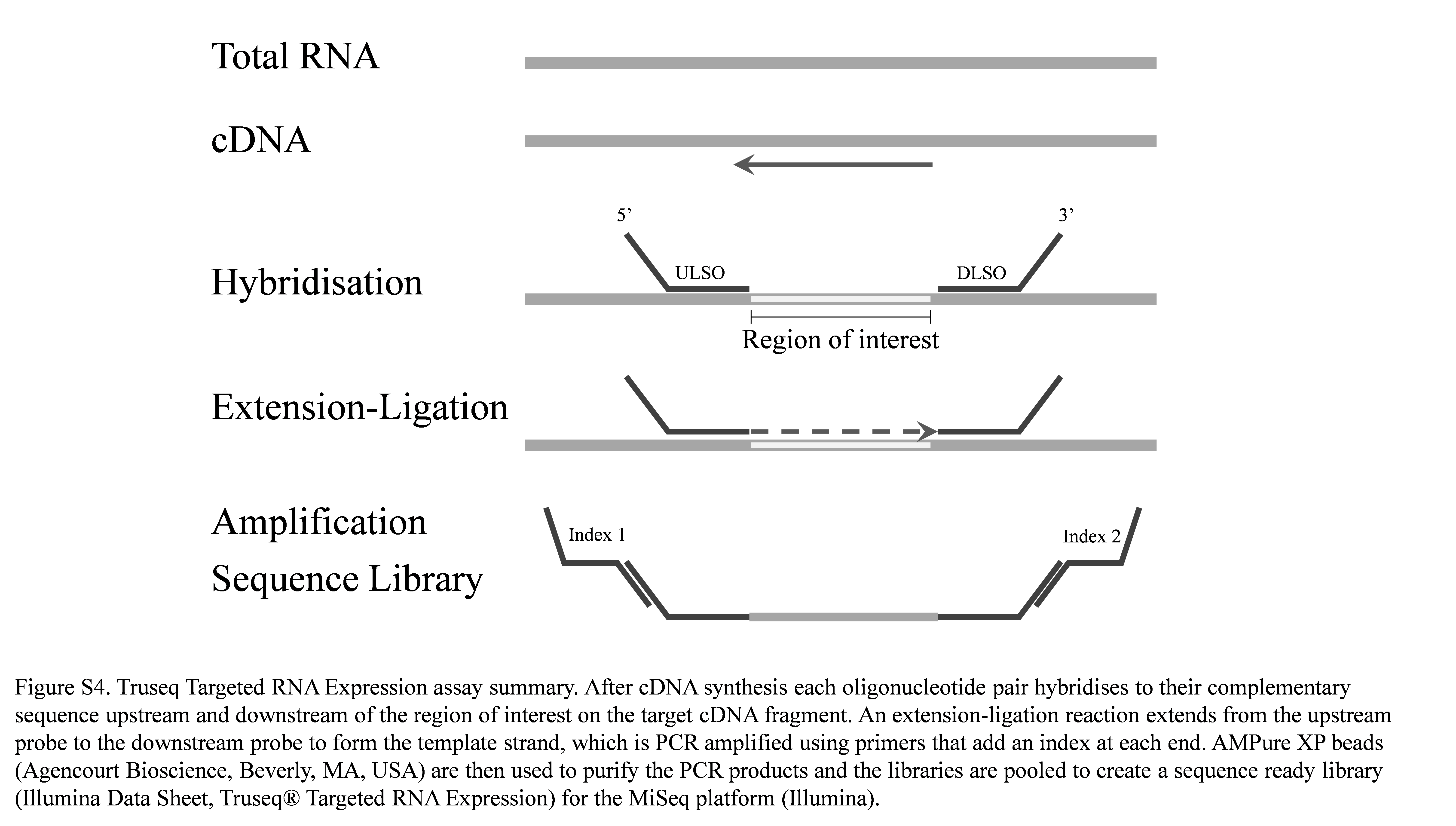

Supplement: Supplementary file 4 [file image_4.tif]

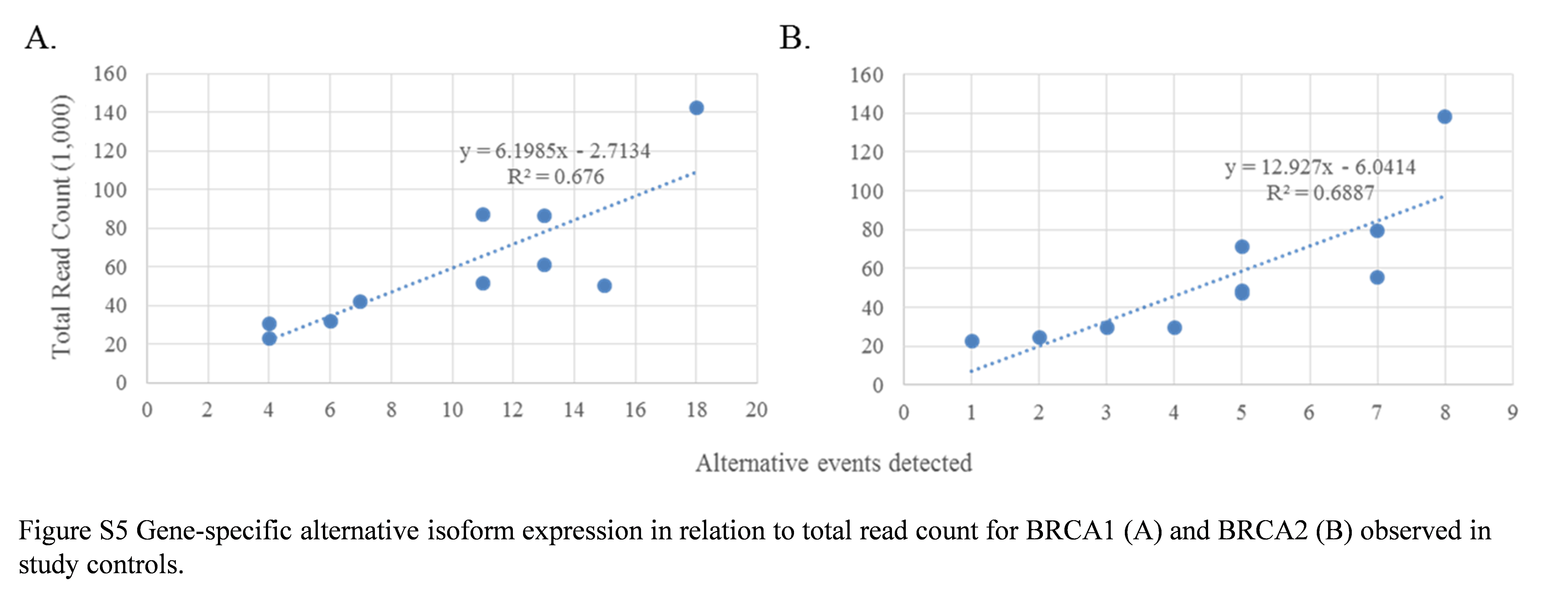

Supplement: Supplementary file 5 [file image_5.tif]

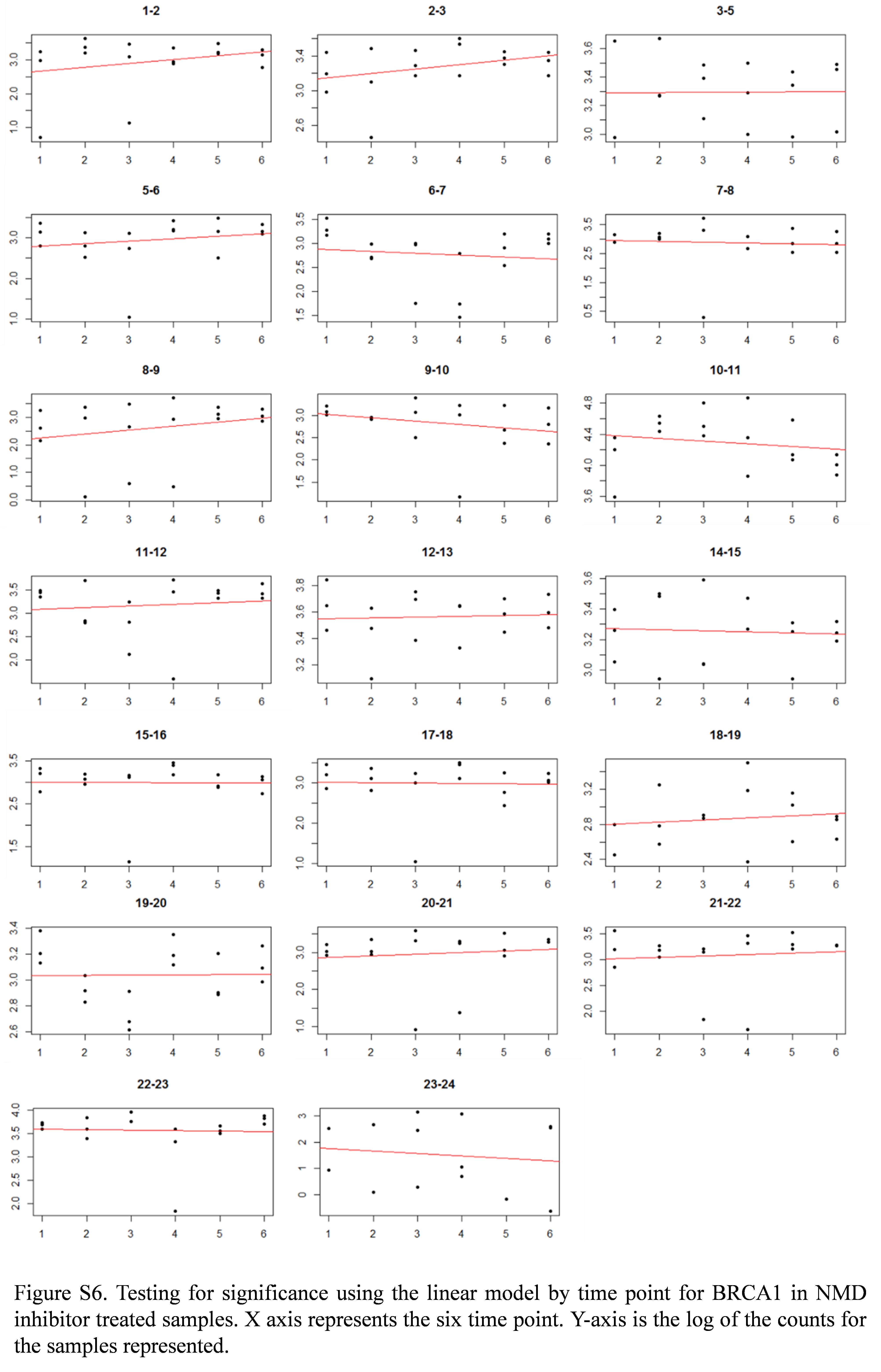

Supplement: Supplementary file 6 [file image_6.tif]

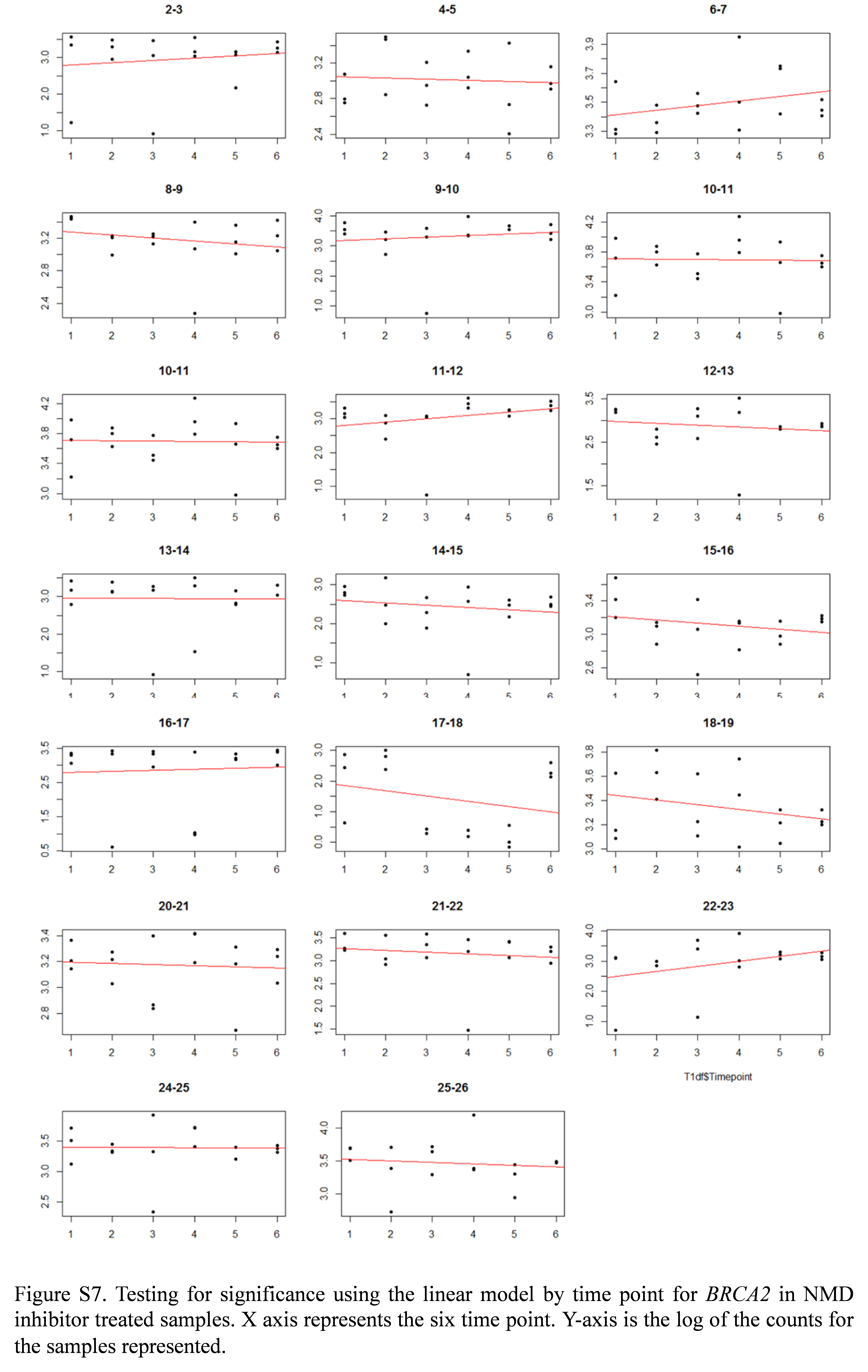

Supplement: Supplementary file 7 [file image_7.tif]

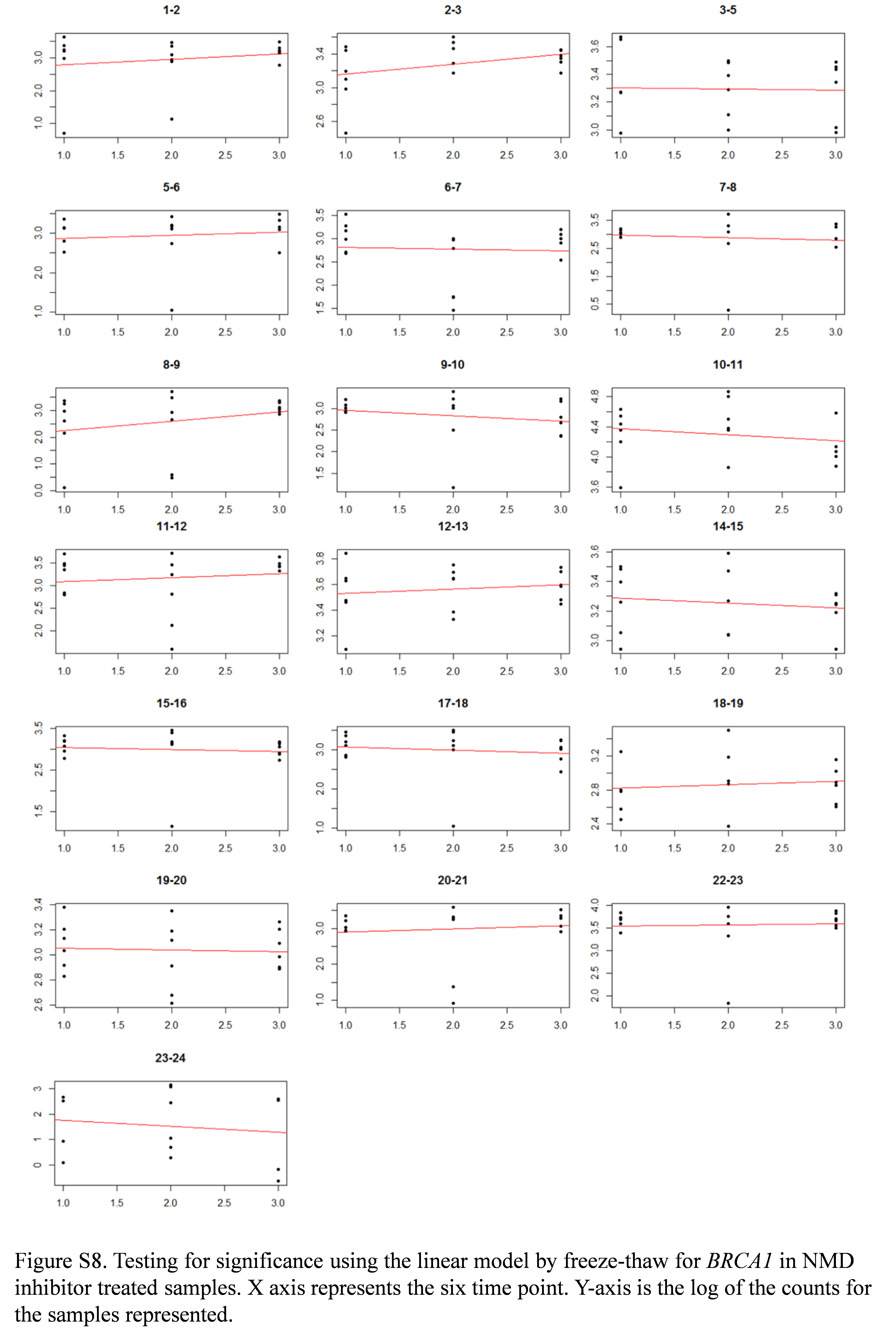

Supplement: Supplementary file 8 [file image_8.tif]

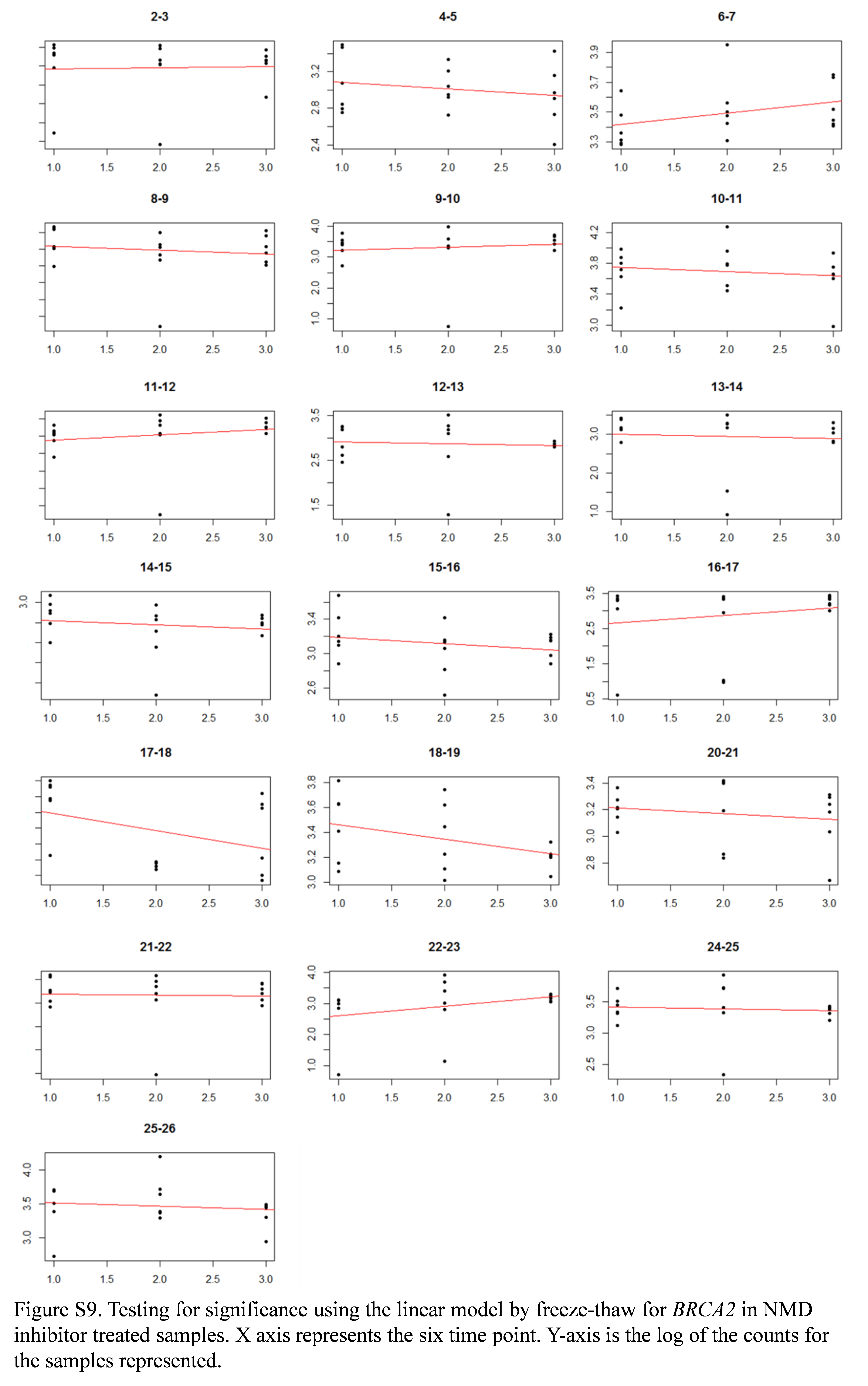

Supplement: Supplementary file 9 [file image_9.tif]

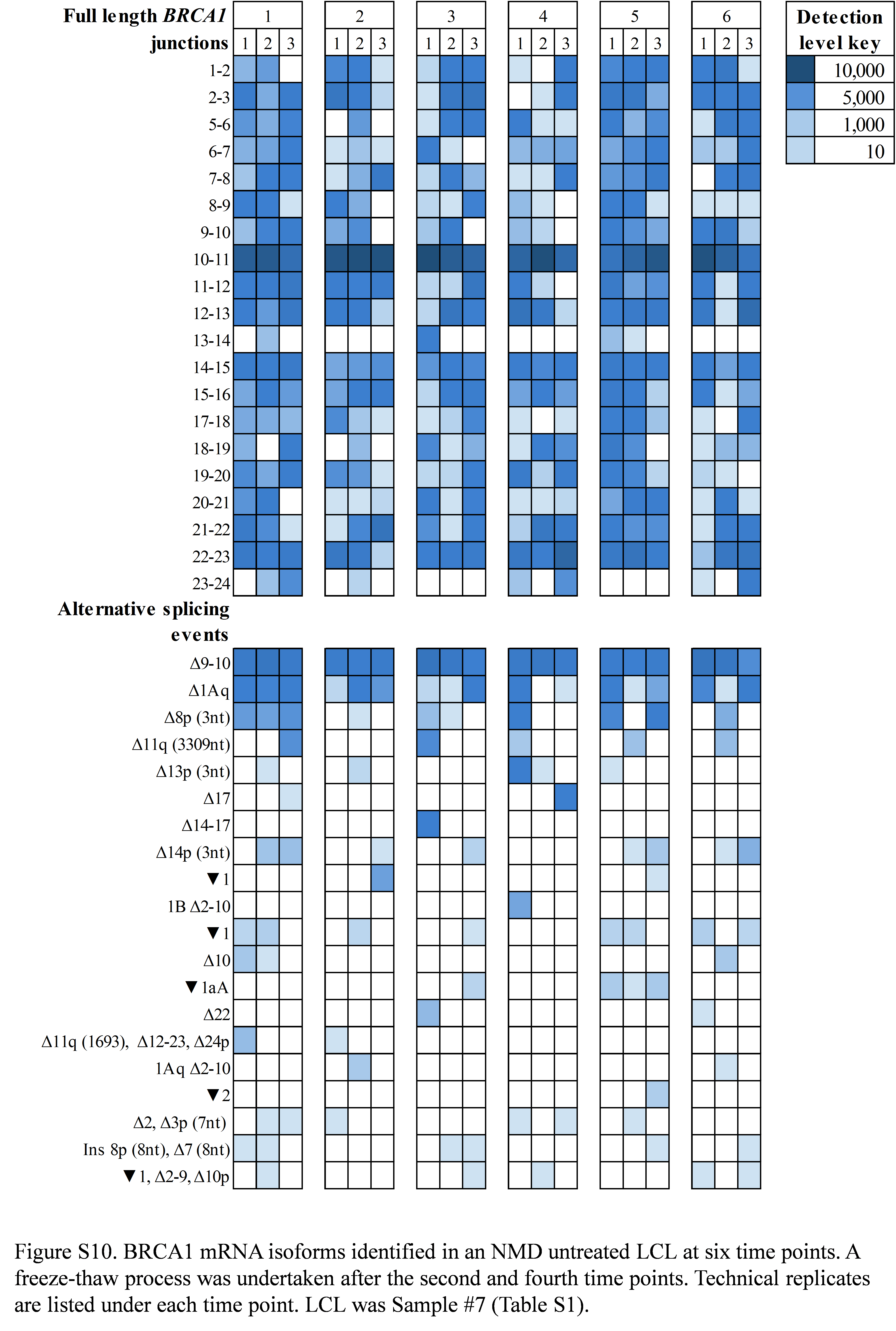

Supplement: Supplementary file 10 [file image_10.tif]

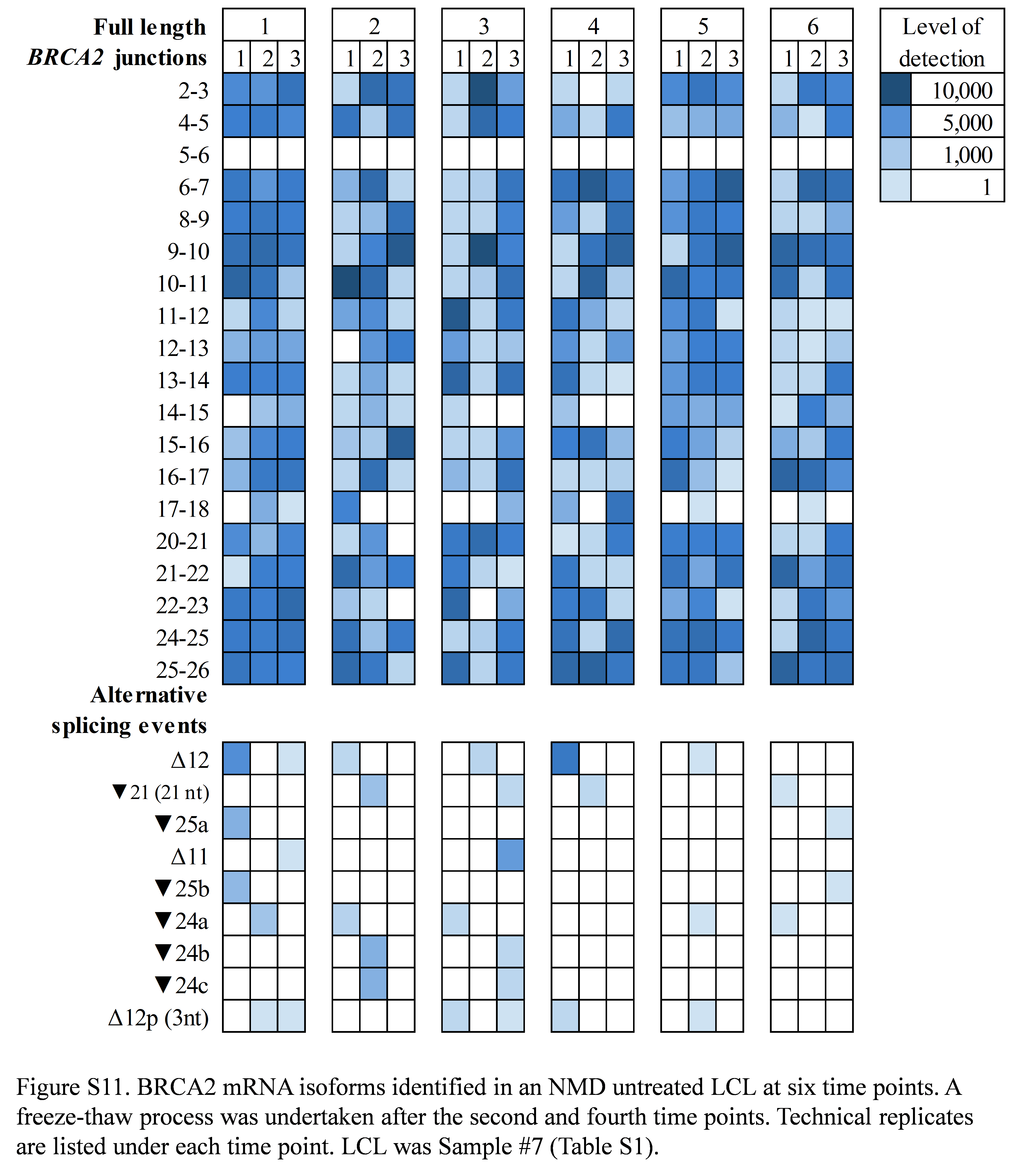

Supplement: Supplementary file 11 [file image_11.tif]
