# Supplementary material for: Investigation of Experimental Factors That Underlie BRCA1/2 mRNA Isoform Expression Variation: Recommendations for Utilizing Targeted RNA Sequencing to Evaluate Potential Spliceogenic Variants
Source: Front Oncol. 2018 May 3;8:140. doi: 10.3389/fonc.2018.00140 (PMC5943536; doi:10.3389/fonc.2018.00140)
Supplement: Supplementary file 12 [file table_1.PDF]

Table S1. *BRCA1* and *BRCA2* rare variant status of samples included in this study.

Sample ID	Variant (BIC nomenclature)	Variant (HGVS nomenclature)	rs ID	BRCAExchange /ClinVar
Rare variant carriers				
LCL1	*BRCA2* IVS 4-12 -8 del5	c.426-12_ 426- 8 delGTTTT	rs27617844	VUS/ Likely pathogenic
LCL2	*BRCA1* IVS 23+5 G>C	c.5467+5 G>C	rs397509287	Unknown
LCL3	*BRCA2* IVS 25+3 A>T	c.9501+3 A>T	rs61757642	Unknown
LCL4	*BRCA1* IVS 10-2 A>G	c.671-2 A>G	rs80358108	Unknown
LCL5	*BRCA1* IVS 9-2 A>C *BRCA1* 760 A>G (D214G)	c.[594-2 A>C; c.641A>G]	rs80358033; rs55680408	Not clinically significant
LCL6	*BRCA1* IVS 4-1 G>T	c.135-1 G>T	rs80358158	Pathogenic
LCL7	*BRCA2* 8216 A>T (E2663V)	c.7988 A>T	rs80359031	Pathogenic
LCL8	*BRCA2* IVS 20+1 G>A	c.8632+1 G>A	rs397507997	Unknown
LCL9	*BRCA1* 2640C>T	c.2521 C>T	rs1800709	Unknown
LCL10	*BRCA1* 2640C>T	c.2521 C>T	rs1800709	Unknown
LCL11	*BRCA1* 2640C>T	c.2521 C>T	rs1800709	Unknown
LCL12	*BRCA1* 2640C>T	c.2521 C>T	rs1800709	Unknown
LCL13	*BRCA1* 2640C>T	c.2521 C>T	rs1800709	Unknown
LCL14	*BRCA1* 2640C>T	c.2521 C>T	rs1800709	Unknown
LCL15	*BRCA1* 2640C>T	c.2521 C>T	rs1800709	Unknown
LCL16	*BRCA1* 2640C>T	c.2521 C>T	rs1800709	Unknown
LCL17	*BRCA1* 2640C>T	c.2521 C>T	rs1800709	Unknown
Controls				
LCL18-27	-	-	-	-

Abbreviations: BIC, Breast Cancer Information Core; HGVS, Human Genome Variation Society; ID, Identifier; IVS, Intervening Sequence; rs, Reference Sequence; VUS, Variant of uncertain significance.
