# Supplementary material for: Investigation of Experimental Factors That Underlie BRCA1/2 mRNA Isoform Expression Variation: Recommendations for Utilizing Targeted RNA Sequencing to Evaluate Potential Spliceogenic Variants
Source: Front Oncol. 2018 May 3;8:140. doi: 10.3389/fonc.2018.00140 (PMC5943536; doi:10.3389/fonc.2018.00140)
Supplement: Supplementary file 15 [file table_4.PDF]

Table S4. Sample specific reads for treated and untreated samples sequenced in this study.

**Sample #**		**NMD inhibitor treated**	**# Sample specific reads**	**Uniquely mapped reads**
1	*BRCA2* c.426-12_8delGTTTT	Yes	143304	95.60%
1	*BRCA2* c.426-12_8delGTTTT	No	146422	95.85%
2	*BRCA1* c.5467+5G>C	Yes	646022	95.91%
2	*BRCA1* c.5467+5G>C	No	431597	96.08%
3	*BRCA2* c.9501+3A>T	Yes	81053	96.07%
3	*BRCA2* c.9501+3A>T	No	98073	95.15%
4	*BRCA1* c.671-2 A>G	Yes	413149	95.95%
4	*BRCA1* c.671-2 A>G	No	21047	95.91%
5	*BRCA1* c.594-2 A>C	Yes	89323	92.23%
5	*BRCA1* c.594-2 A>C	No	83005	95.34%
6	*BRCA1* c.135-1 G>T	Yes	24561	95.41%
6	*BRCA1* c.135-1 G>T	No	30768	95.56%
7	*BRCA2* c.7988 A>T	Yes	22117	94.92%
7	*BRCA2* c.7988 A>T	No	215297	95.98%
8	*BRCA2* c.8632+1G>A	Yes	156715	95.12%
8	*BRCA2* c.8632+1G>A	No	75038	96.18%
9	*BRCA1* 2640 C>T (R841W)	Yes	119655	95.99%
9	*BRCA1* 2640 C>T (R841W)	No	77185	95.51%
10	*BRCA1* 2640 C>T (R841W)	Yes	84107	95.44%
10	*BRCA1* 2640 C>T (R841W)	No	27279	95.62%
11	*BRCA1* 2640 C>T (R841W)	Yes	83196	94.16%
11	*BRCA1* 2640 C>T (R841W)	No	99386	94.89%
12	*BRCA1* 2640 C>T (R841W)	Yes	317435	95.65%
12	*BRCA1* 2640 C>T (R841W)	No	16799	95.81%
13	*BRCA1* 2640 C>T (R841W)	Yes	16395	94.27%
13	*BRCA1* 2640 C>T (R841W)	No	14929	95.72%
14	*BRCA1* 2640 C>T (R841W)	Yes	83642	95.23%
14	*BRCA1* 2640 C>T (R841W)	No	187811	95.45%
15	*BRCA1* 2640 C>T (R841W)	Yes	28771	96.44%
15	*BRCA1* 2640 C>T (R841W)	No	151226	95.74%
16	*BRCA1* 2640 C>T (R841W)	Yes	9066	95.96%
16	*BRCA1* 2640 C>T (R841W)	No	11399	94.64%
17	*BRCA1* 2640 C>T (R841W)	Yes	127966	90.75%
17	*BRCA1* 2640 C>T (R841W)	No	49685	95.30%
18	Control	Yes	110952	94.13%
18	Control	No	67260	97.26%

19	Control	Yes	171318	95.54%
19	Control	No	114396	95.94%
20	Control	Yes	176013	95.98%
20	Control	No	187840	96.04%
21	Control	Yes	140376	96.49%
21	Control	No	103652	95.30%
22	Control	Yes	366409	95.90%
22	Control	No	472421	96.12%
23	Control	Yes	298874	90.08%
23	Control	No	122817	94.60%
24	Control	Yes	542809	95.30%
24	Control	No	37988	95.12%
25	Control	Yes	199225	92.50%
25	Control	No	131922	94.78%
26	Control	Yes	120054	97.75%
26	Control	No	139471	94.46%
27	Control	Yes	271946	95.40%
27	Control	No	155561	94.33%
