# Supplementary material for: Investigation of Experimental Factors That Underlie BRCA1/2 mRNA Isoform Expression Variation: Recommendations for Utilizing Targeted RNA Sequencing to Evaluate Potential Spliceogenic Variants
Source: Front Oncol. 2018 May 3;8:140. doi: 10.3389/fonc.2018.00140 (PMC5943536; doi:10.3389/fonc.2018.00140)
Supplement: Supplementary file 16 [file table_5.PDF]

Table S5. qPCR probes used to validate mRNA expression levels observed in Targeted RNA-seq data.

Assay name		Product	Sequence	Length (bp)	Dye
**FL Exon 2-3**	FWD	PrimeTime Std DNA Primer I	GCTCTTCGCGTTGAAGAAGTA	21	Hex
	REV	PrimeTime Std DNA Primer I	CACACTTTGTGGAGACAGGTT	21	
	PRB	PrimeTime Assay Std Probe 5'	/5HEX/AGAGTGTCC/ZEN/CATCTGTCTGGAGTTGA/3IABkFQ/	26	
**Delta 9-10**	FRD	PrimeTime Std DNA Primer I	TGTGGGAGATCAAGAATTGTTAC	19	FAM
	REV	PrimeTime Std DNA Primer I	CTGGGTTGATGATGTTCAGTATTT	22	
	PRB	PrimeTime Assay Std Probe 5' 6-FAM/ZEN/3' IBFQ	/56-FAM/CCCTCAAGG/ZEN/AACCAGGGATGAAATCA/3IABkFQ/	27	
**FL Exon 10-11**	FRD	PrimeTime Std DNA Primer I	TGTGGGAGATCAAGAATTGTTAC	23	FAM
	REV	PrimeTime Std DNA Primer I	CTGGGTTGATGATGTTCAGTATTT	24	
	PRB	PrimeTime Assay Std Probe 5' 6-FAM/ZEN/3' IBFQ	/56-FAM/CCCTCAAGG/ZEN/AACCAGGGATGAAATCA/3IABkFQ/	26	
**Delta 10**	FWD	PrimeTime Std DNA Primer I	CAACTTATTGCAGCTGCTTGT	21	FAM
	REV	PrimeTime Std DNA Primer I	GCTTCTCAGTGGTGTTCAAATC	22	
	PRB	PrimeTime Assay Std Probe 5' 6-FAM/ZEN/3' IBFQ	/56-FAM/CTGAGACGG/ZEN/ATGTAACAAATACTGAACATC/3IABkFQ/	30	
