# Supplementary material for: Investigation of Experimental Factors That Underlie BRCA1/2 mRNA Isoform Expression Variation: Recommendations for Utilizing Targeted RNA Sequencing to Evaluate Potential Spliceogenic Variants
Source: Front Oncol. 2018 May 3;8:140. doi: 10.3389/fonc.2018.00140 (PMC5943536; doi:10.3389/fonc.2018.00140)
Supplement: Supplementary file 22 [file table_11.PDF]

Table S11. Relative expression analysis of *BRCA1* mRNA junctions using qPCR, with comparison to RNA-seq data.

Sample Name	Sample #	Target	Reference	Ct Target	Ct Control		Target/Ref qPCR	Target/Ref RNA-seq†
BRCA1 c.594-2 A>C	5	Δ 10	FL2-3	28.7	27.37		0.398	0.578
Control	19	Δ 10	FL2-3	35	28.884		0.014	0.077
Control	24	Δ 10	FL2-3	35	27.615		0.006	0.029
Control	20	Δ 10	FL2-3	35	29.439		0.021	0
					**Carrier:controls**		**28.688**	**16.262**
BRCA1 c.594-2 A>C	5	Δ 9-10	FL2-3	27.82	27.248		0.671	1.241
Control	19	Δ 9-10	FL2-3	28.61	27.893		0.607	0.852
Control	24	Δ 9-10	FL2-3	27.92	27.037		0.542	2.38
Control	20	Δ 9-10	FL2-3	29.33	28.595		0.601	1.055
					**Carrier:controls**		**1.15**	**0.869**
BRCA1 c.594-2 A>C	5	FL10-11	FL2-3	28.2	27.435		0.587	2.222
Control	19	FL10-11	FL2-3	27.97	27.84		0.915	7.66
Control	24	FL10-11	FL2-3	27.37	26.724		0.64	4.335
Control	20	FL10-11	FL2-3	28.32	28.158		0.895	3.615
					**Carrier:controls**		**0.718**	**0.427**

†Number of raw reads mapped to the full length exon 2-3 junction compared to the target junction in targeted RNA-seq data.
