# Supplementary material for: Investigation of Experimental Factors That Underlie BRCA1/2 mRNA Isoform Expression Variation: Recommendations for Utilizing Targeted RNA Sequencing to Evaluate Potential Spliceogenic Variants
Source: Front Oncol. 2018 May 3;8:140. doi: 10.3389/fonc.2018.00140 (PMC5943536; doi:10.3389/fonc.2018.00140)
Supplement: Supplementary file 23 [file table_12.PDF]

Table S12. Sample-specific read depth of a single LCL taken at six different time points across three technical replicates. The number of different gene-specific alternative splicing events is listed per sample.

**Time point**	**Technical Rep**	**Total reads**	**# *BRCA1* AS Events**	**# *BRCA2* AS Events**
**Treated**				
1	1	120,775	10	2
1	2	43,192	5	3
1	3	23,364	4	1
2	1	84,758	7	2
2	2	59,229	6	2
2	3	81,529	5	4
3	1	40,564	4	0
3	2	71,409	6	1
3	3	121,723	6	2
4	1	73,117	6	1
4	2	40,702	3	0
4	3	66,563	4	0
5	1	77,598	8	2
5	2	66,451	7	1
5	3	131,536	10	2
6	1	375,445	13	4
6	2	279,706	11	4
6	3	123,599	7	3
**Untreated**				
1	1	53,047	6	2
1	2	96,750	6	0
1	3	29,941	6	0
2	1	16,042	2	0
2	2	57,363	4	1
2	3	11,723	4	0
3	1	15,174	5	0
3	2	10,805	1	0
3	3	61,917	5	0
4	1	39,045	8	1
4	2	19,361	2	0
4	3	15,644	3	0
5	1	38,228	6	0
5	2	64,074	4	0
5	3	37,282	7	0
6	1	32,964	3	0
6	2	46,470	4	0
6	3	69,529	5	0
